# Efficacy and Cytotoxicity of Binary Mixtures as Root Canal Filling Solvents

**DOI:** 10.3390/ma13143237

**Published:** 2020-07-21

**Authors:** Inês Ferreira, Liliana Grenho, Pedro Gomes, Ana Cristina Braga, Maria Helena Fernandes, Maria Ascensão Lopes, Irene Pina-Vaz

**Affiliations:** 1 Faculdade de Medicina, Universidade do Porto, 4200-319 Porto, Portugal; 2 CINTESIS, Faculdade de Medicina Dentária, Universidade do Porto, 4200-393 Porto, Portugal; 3 Laboratory for Bone Metabolism and Regeneration, Faculdade de Medicina Dentária, Universidade do Porto, 4200-393 Porto, Portugal.; lgrenho@fmd.up.pt (L.G.); pgomes@fmd.up.pt (P.G.); mhfernandes@fmd.up.pt (M.H.F.); 4 LAQV/REQUIMTE, U.Porto, 4160-007 Porto, Portugal; malopes@fe.up.pt; 5 Departamento de Produção e Sistemas da Escola de Engenharia, Centro ALGORITMI, Universidade do Minho, 4710-057 Braga, Portugal; acb@dps.uminho.pt; 6 Departamento de Engenharia Metalúrgica e de Materiais, Faculdade de Engenharia da Universidade do Porto, 4200-465 Porto, Portugal

**Keywords:** endodontic material, epoxy resin-based root canal sealer, gutta-percha, retreatment, root canal filling materials, solvents, ultrasonic agitation

## Abstract

Objectives: This study reports the efficacy of two solvent mixtures on the dissolution of gutta-percha and AH Plus sealer, together with the cytotoxicity. Methods: Methyl ethyl ketone (MEK), orange oil, tetrachloroethylene, MEK/tetrachloroethylene (1:1), MEK/orange oil (1:1), and chloroform (control) were tested. Twelve groups (*n* = 15) of standardized stainless-steel molds filled with softened gutta-percha cones and twelve (*n* = 15) filled with AH Plus were immersed in the corresponding mixture or individual solvent, in an ultrasonic bath, for either 2 or 5 min. The effect of the solvents was assessed qualitatively by a topographical analysis (scanning electron microscopy) and chemical analysis (Fourier transform infrared spectroscopy), and quantitatively by a weight loss and viscoelastic property (dynamic mechanical analysis) evaluation. The cytotoxicity was assessed on MG63 human osteoblastic cells. Results: The mixtures did not show the formation of new compounds. Both presented significantly higher efficacies compared to their individual solvents, suggesting a synergistic effect. Their dissolution efficacy was similar to that of chloroform, showing high cytocompatibility. Conclusions: The proposed strategy, incorporating ultrasound agitation and profiting from the synergy of adequate solvents, might enhance root canal cleanliness allowing a single-step procedure to dissolve gutta-percha and the sealer remnants, while assuring cytocompatibility with the periapical tissues.

## 1. Introduction

The prevalence of apical periodontitis is highly correlated with poorly endodontically treated teeth, persisting or emerging as post-treatment apical periodontitis [1,2]. Nonsurgical root canal retreatment is often the first choice to manage secondary endodontic infections. The success of this procedure requires the effective cleaning and subsequent prevention of reinfection, which is only achieved after the complete removal of the old filling materials [3,4]. Gutta-percha does not adhere to the canal walls, and a sealer is recommended in obturation procedures. Although gutta-percha is considered easy to remove, it can persist with sealer remnants entrapped in the dentine structure after retreatment, independently of the filling technique [5]. Micro-CT studies showed that a complete removal of the filling materials is almost impossible, even with the development of new instruments and techniques [6].

The use of endodontic solvents in filling removal, associated with rotary instrumentation, presents controversial results and benefits [7,8,9]. Traditionally, they are applied at the initial stages of retreatment, as a softening filling material. Chloroform and xylene have been generally used; however, concerns regarding their toxicity demanded alternatives [10]. Tetrachloroethylene has some efficacy in dissolving gutta-percha [11,12], zinc-oxide eugenol [13] and resin-based sealers [14] but is less effective than chloroform. Essential oils, including orange oil and eucalyptol, were proposed as biocompatible alternatives, but their dissolution results are not widely accepted [11,14,15]. The use of solvent mixtures has received little attention. Regarding this, the associations of tetrachloroethylene/orange oil and tetrachloroethylene/eucalyptol [11] showed slightly higher effects in the dissolution of gutta-percha and Resilon compared to the essential oils, although lower or similar results were observed compared to tetrachloroethylene. As the cytotoxicity was not evaluated, the real interest of these mixtures remained unclear.

Recently, we reported a new dissolution approach, using a non-traditional endodontic solvent—methyl ethyl ketone (MEK), potentiated by ultrasonic agitation (UA), that seemed an excellent alternative to chloroform in the dissolution of epoxy resin-based sealers (AH Plus) [14]. Additionally, the UA strategy proved to enhance the performance of tetrachloroethylene and orange oil on gutta-percha dissolution, to levels similar to chloroform [12]. Based on these studies we proposed a different approach in cleaning the root canal system by applying specific solvents improved by agitation as an additional final step in retreatment procedures.

Considering the promising outcome of our proposal [12,14] and the poor results of the reported solvent associations, we hypothesized that mixing different solvents has the potential advantage of complementary dissolution, ideally allowing a single-step procedure to dissolve gutta-percha and the sealer remnants, potentiated by agitation in the empty canal while assuring the cytocompatibility with periapical tissues.

In this work, we aimed to explore the dissolution of both the filling material (gutta-percha) and sealer (AH Plus) by two binary mixtures, MEK/tetrachloroethylene and MEK/orange oil, under ultrasonic agitation, compared to the individual solvents, and to assess their cytocompatibility towards osteoblastic cells.

## 2. Materials and Methods

### 2.1. Solvents and Mixtures

MEK, tetrachloroethylene (both, VWR International SAS, Pairs, France), orange oil (Citrol, Biodinamica, Spain), and chloroform (Fisher Scientific UK Ltd., Loughborough, UK), and the mixtures of MEK/tetrachloroethylene (1:1) and MEK/orange oil (1:1) were tested for their efficacy in the dissolution of gutta-percha (Dentsply Maillefer, York, PA, USA) and AH Plus sealer (Dentsply DeTrey, Konstanz, Germany).

Elemental chemical analysis: The potential interactions between the solvents in the mixtures was analyzed by frontier Fourier transform infrared (FTIR) spectrophotometry (PerkinElmer, Villebon-sur-Yvette, France).

### 2.2. Sample Preparation, Dissolution and Characterization

#### 2.2.1. Weight Loss

We fixed 360 standardized stainless-steel molds (diameter, 7 mm; height, 3 mm) on stainless-steel blades and filled them with softened gutta-percha cones (*n* = 180) and AH Plus sealer (*n* = 180) and kept them at 37 °C for 48 h. The gutta-percha samples and the AH Plus samples were each randomly distributed into twelve groups (*n* = 15), which were all maintained at 37 °C for 48 h (IKA KS 4000 ic Control; IKA-Werke GmbH, Staufen, Germany). Then, every mold was weighed on a digital analytical scale before being immersed in the solvents (initial weight). The samples were immersed under agitation in an ultrasonic bath (RETSCH Solutions in Milling & Sieving, Haan, Germany) to a frequency of 30 kHz, at room temperature (RT). Half of the groups were immersed for 2 min and the other half for 5 min in the corresponding solvent or mixture. Then, to neutralize the action of the solvents, the samples were dipped in 10 mL of distilled water for 10 min, blotted dry, and placed again in the incubator for 48 h. Lastly, each mold was weighed again (post-immersion weight). The dissolution of gutta-percha and AH Plus sealer was calculated as a percentage, considering the difference between the initial and the final weight.

This methodology was adapted from the ISO 6876:2012 standard [16] and from other similar studies [11,12,14,17].

#### 2.2.2. Surface Topography

We observed the surface damage and topography of the filling materials (gutta-percha cones and sealer) on the surface exposed to the different solvent conditions (MEK, tetrachloroethylene, orange oil, chloroform, MEK/tetrachloroethylene and MEK/orange oil), using scanning electron microscopy (SEM, Quanta 400FEG SEM, FEI, OR, USA) after sputter coating with gold/palladium (SPI Sputter Coater (SPI Supplies, West Chester, PA, USA)). We observed randomly selected samples, in triplicate, of each endodontic filling material after the weight loss assay.

#### 2.2.3. Mechanical Properties

We analyzed the mechanical properties of gutta-percha cones after a 5 min immersion in the different solvent conditions tested (MEK, tetrachloroethylene, orange oil, chloroform, MEK/tetrachloroethylene and MEK/orange oil), with dynamic mechanical analysis (DMA Tritec 2000, Triton Technology Ltd., Nottingham, UK (single cantilever bending mode, 1 Hz frequency, 37 °C)). The AH Plus sealer lost its structure after immersion, preventing its analysis. Triplicate samples of each condition were analyzed.

### 2.3. The Cytotoxicity of Solvents and Mixtures

MG63 human osteoblastic cells (ATCC^®^ CRL-1427) were cultured in α-minimum essential medium (α-MEM), 10% fetal bovine serum, 100 U/mL penicillin/100 µg/mL streptomycin and 2.5 µg/mL amphotericin B (all from GIBCO), at 37 °C and 5% CO_2_. The cells (104/cm^2^) were cultured for 48 h (~70% confluence), washed with phosphate-buffered saline (PBS, Sigma-Aldrich, St. Louis, MO, USA) and, then, incubated for 5 min at 37 °C in the testing solutions, at 1:1, 1:10 or 1:20 dilutions (prepared in α-MEM). Then, the cultures were stained with propidium iodide (PI, 50 µL/mL, BD Biosciences) and calcein-AM (1 µM, BioLegend, San Diego, CA, USA) and observed (Celena S digital imaging system, Logos Biosystems, Anyang-si, Korea). Fifteen random images from each condition (isolated solvents and mixtures as well as chloroform) were analyzed with ImageJ (version 1.8, NIH, Bethesda, MD, USA), to determine the live/dead cell percentages. The results were compared to those from control cultures (not exposed to the solvents).

### 2.4. Statistical Analysis

The statistical differences between the groups were analyzed using nonparametric tests such as the Mann–Whitney (MW) test for two independent groups and the Kruskal–Wallis (KW) test for more than two independent groups, followed by a post hoc test when a significant difference was detected. The significance level was set at *p* < 0.05. All statistical procedures were conducted in IBM SPSS 26.0 software (SPSS Inc., Chicago, IL, USA).

## 3. Results

### 3.1. Elemental Chemical Analysis of the Solvents and Mixtures

**Figure 1:**
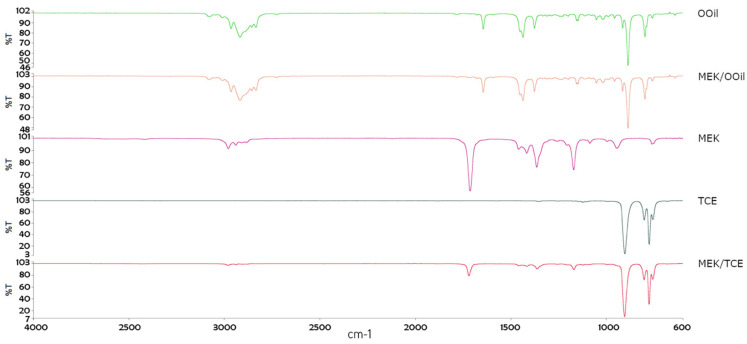
Frontier Fourier transform infrared (FTIR) spectroscopic plots of the isolated solvents and binary mixtures—OOil, orange oil; MEK/OOil; MEK, methyl ethyl ketone; TCE, tetrachloroethylene; MEK/TCE.

The solvents were analyzed using FTIR (Figure 1). The MEK spectrum revealed a saturated ketone (intense bands at 1713, 2940, 1365 and 1170 cm^−1^, attributable to C=O, C–H stretches, CH3C=O bending and a C–CO–C stretch and bend, respectively). The tetrachloroethylene spectrum showed the vibration of C–Cl bonding at ~775 cm^−1^. Essential oils, primarily composed of terpenes [18], revealed signals in the 2900–3100 cm^−1^ range attributable to the asymmetrical and symmetrical stretching vibrations of C–H, at ~1700 cm^−1^ for a C=O stretch, and at ~1100 cm^−1^ for a C–O stretch. In binary mixtures, the characteristic bands of MEK were strongly attenuated, despite no new bands being identified, indicating that no chemical interaction occurred between the mixed solvents.

### 3.2. Dissolution of Gutta-Percha and AH Plus Sealer

#### 3.2.1. Weight Loss

**Figure 2:**
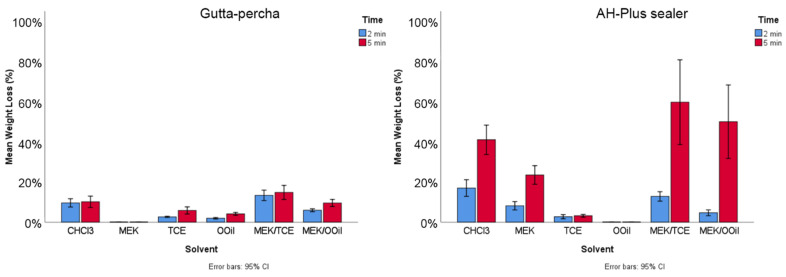
The weight loss of the gutta-percha and AH Plus samples after immersion in the tested solutions for 2 and 5 min—CHCl_3_, chloroform; MEK, methyl ethyl ketone; TCE, tetrachloroethylene; OOil, orange oil. Mixtures: MEK/TCE; MEK/OOil.

Statistical analyses detected significant differences between the materials (MW: Z = −4.068, *p* < 0.05), solvents (KW: χ^2^(5) = 189.862, *p* < 0.05) and exposure time (MW: Z = −4.487, *p* < 0.05). The multiple comparison tests (Table 1) and the graphs of Figure 2 revealed that chloroform, MEK and the two mixtures caused a significantly higher weight loss in the AH Plus samples compared with the gutta-percha samples. Dissolution was significantly higher in the AH Plus sealer compared to gutta-percha (MW: Z = −4.068, *p* < 0.05).

Both mixtures presented higher dissolution values than the individual solvents (*p* < 0.05). At a 5 min exposure, the effect was similar for both mixtures to that of chloroform. The dissolution of AH Plus was time-dependent (*p* < 0.05), unlike for gutta-percha. The results are presented in Figure 2.

#### 3.2.2. Surface Topography

**Figure 3:**
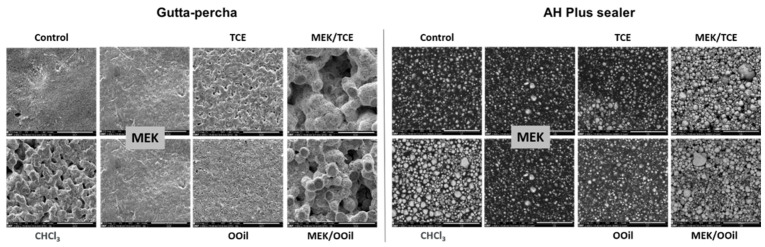
Representative scanning electron microscopy (SEM) images of the gutta-percha (secondary electron mode) and AH Plus (backscattered electron mode) samples before (control) and after 5 min of immersion in the tested solutions—CHCl_3_, chloroform; MEK, methyl ethyl ketone; TCE, tetrachloroethylene; OOil, orange oil. Mixtures: MEK/TCE; MEK/OOil. Bar = 50 µm.

Figure 3 shows the representative SEM images of the sample surfaces, after a 5 min immersion, under ultrasonic agitation. The gutta-percha samples were observed in the secondary electron mode, allowing for topography information. The control samples (not exposed to the solvents) and MEK-treated samples displayed a relatively smooth surface compared to the significant dissolution features observed with chloroform, followed by tetrachloroethylene and orange oil. Comparatively, both mixtures presented a higher dissolution efficacy, evidenced by the greatly damaged surface topography.

The AH Plus samples were analyzed in the backscattered electron mode, to detect the contrast between areas rich in light (e.g., carbon) or heavy (e.g., zinc or tungstate) atoms, appearing darker or lighter, respectively. The control samples exhibited a dark appearance with clear granular structures. Chloroform caused a significant decrease in the dark areas, suggesting the dissolution of the carbon-rich organic components, and the surface predominantly showed a lighter granular appearance of the heavy metals (with higher atomic numbers) present in the composition. The mixtures exhibited similar behaviors. The other solvents had little effect.

#### 3.2.3. Mechanical Properties

**Figure 4:**
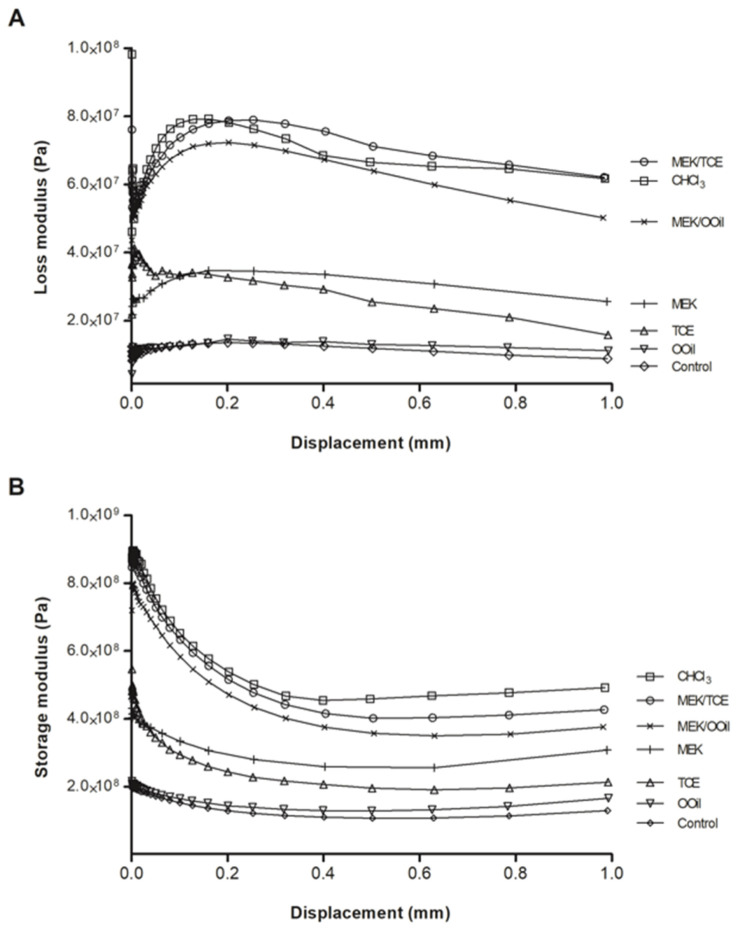
The storage modulus (**A**) and loss modulus (**B**) of the gutta-percha samples after immersion in the tested solutions for 5 min—control (samples not exposed to the solvents); CHCl_3_, chloroform; MEK, methyl ethyl ketone; TCE, tetrachloroethylene; OOil, orange oil. Mixtures: MEK/TCE; MEK/OOil.

Analysis of the viscoelastic properties of gutta-percha, Figure 4, showed that chloroform caused the highest stiffness (highest storage modulus), followed by the binary mixtures, MEK and, then, the other solvents. The variation of the loss modulus followed a similar trend.

### 3.3. In Vitro Cytotoxicity

**Figure 5:**
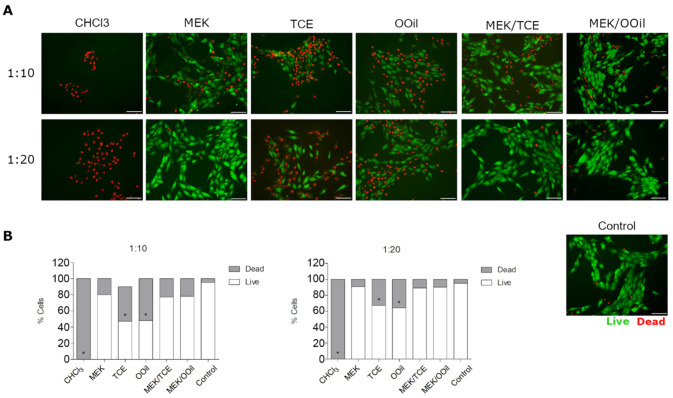
The cell viability/survival of MG63 osteoblastic cells after exposure to the tested solutions, as determined by fluorescent-based live-dead cell staining. (**A**) Fluorescence images of live (green) and dead (red) cells (scale bar: 100 µm); (**B**) percentage of live and dead cells. * Significantly different from control (cultures not exposed to the solvents), *p* < 0.05.

Figure 5 presents the results for the live/dead assay, performed in MG63 osteoblastic cells. The control cultures (incubated only with culture medium) showed high cell survival, with rare events of dead cells. All tested solvents and mixtures caused cell death at a 1:1 dilution (data not shown), as well as chloroform at 1:10 and 1:20 dilutions (*p* < 0.05). MEK and the binary mixtures presented the lowest cytotoxicity, i.e., cell survival was ~80% and ~90% at the 1:10 and 1:20 dilutions, respectively, showing no significant differences to the established control. Incubation with tetrachloroethylene or orange oil showed improved viability compared to chloroform (~50% and 70% of live cells, respectively at the 1:10 and 1:20 dilutions), although this was significantly inferior to that of the control (cultures not exposed to the solvents (*p* < 0.05)).

## 4. Discussion

This study evaluated the dissolution efficacy of the mixtures of MEK/tetrachloroethylene and MEK/orange oil on gutta-percha and AH Plus sealer, under ultrasonic agitation, compared to that of the individual solvents. FTIR spectroscopy (Figure 1) ensured that none of the mixtures contained new compounds other than the solvents initially associated. Thus, the results were compared to the individual solvents and with chloroform, which is the most used solvent due to its efficacy, although there are associated safety concerns [19,20].

AH Plus sealer and gutta-percha comprise organic polymeric matrices with embedded inorganic components [21,22,23]. Endodontic solvents interact mainly with the organic matrix aiming to dissolve one or several components disturbing the spatial structure and increasing the effectiveness of the material removal. The solvent profiles (i.e., polarity) and the fillings’ features (i.e., the organic phase composition, polymeric matrix type, and crystallinity) and organic/inorganic phases’ interactions determine the dissolution efficacy. In this work, MEK, tetrachloroethylene and orange oil presented lower performances when compared to chloroform. By contrast, the proposed mixtures caused similar or higher dissolution features as those observed with chloroform, a result not previously reported.

Concerning the two materials, AH Plus sealer exhibited a higher weight loss compared to gutta-percha. The manufacturing route of the latter produces a semi-crystalline material of gutta-percha molecules in the form of folded chains maintained by secondary bonds and crosslinked zinc oxide, posing a high resistance to the structural and chemical etching of the organic phase [21,24].

MEK was selected to be the common solvent in the two proposed mixtures, based on its reported efficacy in dissolving AH Plus sealer [14], a result confirmed in the present work. However, MEK had little effect on gutta-percha as shown here. When mixed with tetrachloroethylene and orange oil, both caused a low weight loss for gutta-percha and an even lower weight loss for AH Plus sealer (Figure 2). The mixtures exhibited a significantly increased efficacy compared to their respective individual solvents, causing effects similar to chloroform on gutta-percha and on AH Plus sealer, as shown by the weight loss results and SEM observations. This clearly suggests a synergistic effect of the involved solvents, which did not result from the formation of new compounds, as proven by the FTIR analysis. As the dissolution efficacy depends on the solvents and filling material’s features, it is likely that one of the solvents in the mixture etches/degrades particular components of the organic phase preferentially, creating weakened localized areas or even channels through the matrix that favor an interaction with the other solvent, greatly enhancing the dissolution outcome. In the case of gutta-percha, MEK had a negligible effect, thus tetrachloroethylene or orange oil (depending on the mixture) would be the determinant to initiate the interaction with the matrix, triggering/allowing MEK interaction. Concerning the AH Plus sealer, MEK had a higher efficacy compared to tetrachloroethylene or orange oil; thus, MEK should play a role in the dissolution onset. Mixing solvents with different profiles may trigger a dynamic chemical environment, greatly enhancing the overall dissolution process. This positive outcome is expected to have greater significance in materials that are easier to disintegrate or dissolve, explaining the better dissolution rate of AH Plus sealer and, also, the time-dependent effect, comparatively to gutta-percha. Additionally, as a whole, the efficacy of the mixtures clearly depended on the dissolution profile of the individual solvents.

Cytocompatibility is a safety requirement for the root canal irrigants [25]. These solvents can easily reach the periapical tissues and affect the behavior of the bone cells, disturbing the local bone metabolism and hampering the healing process. The two proposed mixtures, in addition to the high dissolution performance, had a high cytocompatibility with human osteoblastic cells, in contrast with the very high cytotoxicity of chloroform, and they also presented a safer proposal than the isolated solvents.

Damage to the root dentine walls, which is observed with highly potent solvents, such as chloroform or xylene, is not expected due to the lower aggressive profile of the solvents included in these mixtures [26,27,28].

The present study has some limitations. The anatomic variations and the close-end in vivo channel are not reproducible in the methodology described here. However, the results point to the interest in performing ex vivo studies to further address this issue.

## 5. Conclusions

To conclude, the highly cytocompatible binary mixtures, MEK/tetrachloroethylene and MEK/orange oil, in association with ultrasonic agitation, proved to be an effective strategy to simplify and enhance the dissolution process with a single solution being effective for both filling materials. Their specificity, potentiated by agitation and the different purpose of removing remnants, instead of only softening, might bring a new perspective on the utility of endodontic solvents. The use at a later stage of the retreatment, as an adjunctive procedure, after the removal of the bulk of filling materials, might be a further step to improve retreatment outcomes.

## Figures and Tables

**Table 1:** Pairwise comparisons of solvent for weight loss after the Kruskal–Wallis test.

Sample 1	Sample 2	*P* ^a^
Orange Oil	Tetrachloroethylene	0.143
Orange Oil	MEK	0.002
Orange Oil	MEK/Orange Oil	<0.001
Orange Oil	Chloroform	<0.001
Orange Oil	MEK/Tetrachloroethylene	<0.001
Tetrachloroethylene	MEK	1.000
Tetrachloroethylene	MEK/Orange Oil	<0.001
Tetrachloroethylene	Chloroform	<0.001
Tetrachloroethylene	MEK/Tetrachloroethylene	<0.001
MEK	MEK/Orange Oil	0.003
MEK	Chloroform	<0.001
MEK	MEK/Tetrachloroethylene	<0.001
MEK/Orange Oil	Chloroform	0.141
MEK/Orange Oil	MEK/Tetrachloroethylene	0.014
Chloroform	MEK/Tetrachloroethylene	1.000

Each row tests the null hypothesis, i.e., that the Sample 1 and Sample 2 distributions are the same. Asymptotic significances (2-sided tests) are displayed. The significance level is 0.05. ^a^—Significance values have been adjusted by the Bonferroni correction for multiple tests.
